# Acceptance and commitment therapy adapted for women with infertility: a pilot study of the Infertility ACTion program

**DOI:** 10.1186/s12978-024-01766-5

**Published:** 2024-04-04

**Authors:** Ashley A. Balsom, Bridget Klest, Bethany Sander, Jennifer L. Gordon

**Affiliations:** Department of Psychology, University of Regina, Regina, SK Canada

**Keywords:** Infertility, Feasibility, Acceptance and Commitment Therapy, Infertility-related distress

## Abstract

**Background:**

Approximately one in six couples are currently infertile, defined as unable to achieve pregnancy despite 12 or more months of active attempts to conceive. Experiencing infertility has been disproportionately associated with an array of psychological difficulties, particularly in women. However, currently available psychological interventions have had minimal benefits for distress, anxiety, or depression related to infertility.

**Methods:**

A one-arm pilot study was conducted to test the acceptability of a newly created acceptance and commitment therapy-based self-guided program—Infertility ACTion. Twenty women, located in Canada, completed the program and completed measures assessing expectancy of improvement, treatment credibility, participant satisfaction, treatment completion and retention, psychological flexibility, fertility quality of life, depression, and anxiety. Participants were also asked to provide feedback on how the researchers could improve the intervention. Paired sample t-tests were conducted to compare pre- and post-intervention outcomes.

**Results:**

Sixteen out of 20 participants completed the entire intervention. Reported treatment expectancy, credibility and satisfaction were favorable. Eighty-one percent of participants reported that they would recommend the program to a friend and 88% thought the program was worth their time. Medium increases in psychological flexibility and fertility quality of life were observed. Improvements in anxious and depressive symptoms were in the small to medium range but were not significant. Participants had several recommendations for program improvement.

**Conclusions:**

This acceptance and commitment therapy-based self-guided program proved to be an acceptable treatment for infertility-related distress. Participant feedback will be used to adjust the current intervention in preparation for a more rigorous randomized-controlled trial testing this program.

**Supplementary Information:**

The online version contains supplementary material available at 10.1186/s12978-024-01766-5.

## Introduction

Infertility, defined as an impairment of a person’s capacity to reproduce as an individual or with a partner, affects approximately 13% of the population [1, 2]. In cisgendered heterosexual couples, women^1^ have been found to experience more of the psychological burden, perhaps because the onus to seek information and undergo fertility testing and treatment tends to be on the intended pregnant partner [3, 4]. In fact, half of infertile women describe infertility as the most upsetting event of their life whereas only 15% of their male partners do [5]. One-third of women undergoing medically assisted reproduction meet criteria for an anxiety disorder or major depressive disorder [6, 7]. Attempting to conceive without medical intervention is associated with similar rates of psychopathology [8, 9]. While the psychological burden of infertility is well documented, currently available psychological interventions have proven largely ineffective. The most recent meta-analysis of psychological interventions for infertility identified 58 randomized controlled trials and found that such interventions have only small effects on infertility-related distress [10]. Efficacy did not differ according to intervention type or format. These findings highlight a need for efficacious mental health interventions for this population.

Acceptance and Commitment Therapy (ACT) is considered part of the ‘third-wave’ of cognitive behaviour therapies. While rooted in traditional cognitive behavioural approaches, ACT incorporates a focus on other skills, particularly mindfulness, more than traditional approaches. The goal of ACT is to encourage *psychological flexibility* by exploring one’s relationship with one’s thoughts, feelings, memories, and physical sensations. Psychological flexibility is defined as the ability to be in contact with private experiences (i.e., thoughts and feelings) in the present moment while behaving in a way that is consistent with one’s values [11]. In other words, psychological flexibility reflects an individual’s ability to live life to the fullest despite mental health symptoms like low mood or anxiety. Though ACT has modest support as a treatment for obsessive–compulsive disorder, depression, anxiety, and psychosis, according to the American Psychological Association (APA) division 12, it has strong support for treating mental health problems associated with chronic or persistent pain [12]. ACT for chronic pain largely aims to encourage individuals to focus less on their pain and to focus more on engaging in activities that are meaningful to them despite their pain. Such an approach may translate well to infertility-related distress; infertility has been associated with a narrowed focus on trying to get pregnant and a decreased involvement in previously-enjoyed activities [8, 13], similar to chronic pain. For example, a woman experiencing infertility may avoid spending time with her friend who is pregnant, or stop working out because of fears that exercise may negatively impact fertility.

To date, there have been only three studies from the Middle East testing the use of ACT for individuals experiencing infertility, with promising results [14–16]. The interventions ranged from 8 to 20 sessions and were delivered via individual and group therapy formats [14–16]. In North America, there has been one case study reporting success in using ACT in helping a distressed couple experiencing infertility [17]. However, there have not been any self-administered ACT interventions for infertility. Self-administered mental health interventions have numerous advantages over traditional in-person formats, including increased accessibility and decreased cost. Furthermore, recent meta-analytic evidence suggests that self-help mindfulness-based interventions are beneficial for depression, anxiety, stress, and quality of life in public health settings [18]. Our team therefore aimed to develop and pilot a brief ACT-based self-help mental health intervention for women experiencing distress related to infertility.

The current study served as a one-arm feasibility study. Treatment feasibility was assessed via a number of indicators, including expectancy of improvement, treatment credibility, participant satisfaction, participant completion and retention, and changes in psychological flexibility, fertility quality of life, depression, and anxiety. Participants were also asked to provide feedback on how the researchers could improve the intervention.

## Methods

### Intervention development

Six modules were created—one to cover each of the six core processes in ACT: acceptance, cognitive defusion, present moment focus, self as context, values, and committed action. A script was drafted for each module by AAB and reviewed and edited by BK, JLG, and two female patient advisors who have lived experience with infertility. These scripts were then turned into a video by pairing a PowerPoint presentation with voiceover. In order to maximize participant adherence, especially considering that many participants would be undergoing burdensome fertility treatments, the research team aimed to keep each of the modules under 15 min long, with homework to be completed between modules. In creating the modules, several ACT-based manuals were consulted [19, 20]. The content was adapted to infertility using the research team’s knowledge of the psychology of infertility, the patient advisors’ input, and consulting the case study by Peterson and Eifert [15] for inspiration. The finished product was reviewed and approved by the team’s patient advisors. A brief description of each of the modules is provided in Table 1.

**Table 1:** Summary of intervention module

Module	Focus
1. Values	Encourages participant to reflect on their life values and consider how their daily actions align with those values. The participant completes an exercise aimed at identifying values that may be currently neglected, potentially due to challenges associated with infertility. This module introduces the concept of “choice point”, which is a moment in time when one must choose between a behaviour that is consistent with one’s values and one that is inconsistent. Examples include calling one’s pregnant sister and deciding whether or not to attend a friend’s baby shower. Homework: Try 80th birthday exercise
2. Cognitive Defusion	Introduces the idea that we have little control over our thoughts and that it is counter-productive to try to stop ourselves from having unpleasant thoughts and emotions. Instead, the participant is encouraged to make space for unpleasant thoughts, such as “I will never get pregnant” or “my partner deserves someone that can give them a child”—by allowing them to simply occur, we are giving them less power than if we were to expend energy trying to stop them. Homework: Practice using daily cognitive defusion strategies (e.g., thanking your mind, using external voice)
3. Present Moment Focus	Introduces the concept of “mindfulness” and has the participant practice being focused on the present moment and identify instances when they might struggle being in the moment. An example is given of an individual being preoccupied about a call they’re expecting from their fertility clinic and having difficulty enjoying a meal with their partner. The analogy of examining one’s thoughts like leaves on a stream is given. Homework: Listen to mindfulness recording daily
4. Acceptance and Willingness	Emphasizes the idea that pain is an inevitable part of life and that accepting one’s pain can decrease the suffering associated with that pain. If we are willing to experience pain, we can live our lives again because we no longer have to avoid situations that are potentially unpleasant (e.g., family get-together with children present). Homework: Practice mindfulness when experiencing distressing thoughts or emotions
5. Self as Context	Encourages the participant to reflect on how their experience with infertility has influenced their view of themselves. They are encouraged to consider that they are still the same person despite all that events occurring around them (e.g., pregnancy loss, infertility, family members not understanding). The aim is to foster a more nuanced understanding of one’s identity that incorporates all of their experiences. Homework: Listen to “continuous you” recording once a day
6. Committed Action/ wrap up	Provides an overview of the program’s content and encourages the individual to reflect on what they’ve accomplished as well as areas for further development. Revisits the concept of avoidance (e.g., withdrawing from friends and family, avoiding children and pregnant women) and encourages the individual to consider ways to reduce avoidance without worsening their distress. Homework: Reflect on their progress in the program and identify how they want to continue to work towards their values

The Infertility ACTion Program was designed to be a self-help intervention paired with scripted emails from the research team. Participants received a module each week via email on a day chosen by them. Two days after the module was sent, they were sent a second email reminding them to watch the module video and assigning homework for that week. Finally, an online survey was sent at the end of each week, requesting that participants report the total number of minutes spent engaging in the assigned homework.

### Participants

Participants were recruited through online infertility support groups throughout Canada. Individuals were deemed eligible if they had been attempting to conceive for 12 or more months or were currently undergoing medically assisted reproduction and were the intended gestational carrier. Eligible participants had to endorse at least a moderate amount of distress, which was defined as a score below 52 on the Fertility Quality of Life scale [21]. Participants were ineligible if they scored seven or above on the Suicide Behaviours Questionnaire–Revised, a score range that has been found to have a sensitivity of 0.93 and a specificity of 0.95 in the identification of suicide risk in adult populations [22]. Participant recruitment began on June 22nd, 2022, and finished July 25th, 2022.

### Procedure

Individuals found to be eligible completed a Zoom-facilitated enrollment session confirming participants’ eligibility for the study, providing information about the project, and obtaining informed consent. Participants completed the pre-intervention, weekly, and post-intervention measures.

### Pre-intervention measures

#### Demographic and reproductive health history questionnaire

Participants were asked to report demographic information and to complete questions about their reproductive health.

#### Patient Health Questionnaire-9 (PHQ-9; [23])

The PHQ-9 is a 9-item questionnaire based on DSM-IV criteria for depressive disorders. Items on the PHQ-9 are on a 4-point Likert scale ranging from 0 (*Not at all*) to 3 (*Nearly every day*). The internal consistency for the current study was Cronbach’s alpha (α) = 0.74.

#### Generalized Anxiety Disorder-7 (GAD-7; [24])

The GAD-7 is a 7-item questionnaire based on DSM-IV criteria for generalized anxiety disorder. Items on the GAD-7 are on a 4-point Likert scale ranging from 0 (*Not at all*) to 3 (*Nearly every day*). The current study demonstrated good internal consistency (Cronbach’s alpha (α) = 0.86).

#### Fertility Quality of Life Questionnaire (FertiQoL; [25])

The FertiQoL is a 36-item questionnaire. The questionnaire focuses on three areas of quality of life: core quality of life, treatment-related quality of life and life and physical health quality of life. Items are on a 5-point Likert scale ranging from 0 (*Completely, Very Dissatisfied, Always, An Extreme Amount*) to 4 (*Not at all, Very Satisfied, Never, Not at All*). The Core FertiQoL had an internal consistency of Cronbach’s alpha α = 0.78 with the subscales ranging from Cronbach’s alpha α’s = 0.60-0.86.

#### Revised Dyadic Adjustment Scale (RDAS [26])

The RDAS is a 14-item measure of the quality and adjustment of a couple’s relationship. The RDAS provides three subscales, including the Dyadic Consensus Subscale, Dyadic Satisfaction Subscale, and Dyadic Cohesion Subscale. It has been found to have good construct validity with both distressed and non-distressed couples [27]. In the current sample, internal consistency was α = 0.88.

#### Multidimensional Psychological Flexibility Inventory (MPFI [28])

The MPFI is a 60-item measure of psychological flexibility. The MPFI examines 12 dimensions of flexibility and inflexibility inspired by the hexaflex model of ACT. The MPFI explores psychological flexibility (acceptance, present moment awareness, self as context, defusion, values, committed action) and inflexibility (experiential avoidance, lack of contact with the present moment, self as content, fusion, lack of contact with values, and inaction). The internal consistency of the MPFI was α = 0.84 for flexibility and α = 0.83 for inflexibility.

#### The Credibility/Expectancy Questionnaire (CEQ [29])

It is a 6-item measurement of credibility and expectancy with items ranging from 1–9 or 0–100%. The CEQ has been found to have good interrater consistency and test–retest reliability [29].

All measures were scored consistent with published recommendations for scoring (e.g., FertiQoL Scoring https://sites.cardiff.ac.uk/fertiqol/scoring/). They were scored using syntax in SPSS version 28.0.

### Weekly measures

Each week, participants rated their depressive via the PHQ-9, anxious symptoms via the GAD-7, and infertility-related quality of life via the FertiQoL. Participants also rated how helpful they found that week’s module and that week’s homework assignment in helping them cope with their infertility struggles on a scale from 0 to 10 and also reported the number of minutes spent engaged in homework over the past week. An open-ended question also asked to provide feedback about the content of the module, including aspects they found helpful or suggestions for improvement.

### Post-intervention measures

The post-intervention measures consisted of the previously administered pre-intervention measures (i.e., PHQ-9, GAD-7, FertiQoL, RDAS, MPFI) in addition to the Treatment Satisfaction Questionnaire, Adverse Effects Questionnaire and open-ended questions about the overall program and suggestions for improvement.

#### Treatment Satisfaction Questionnaire (TSQ [30])

The TSQ was used to assess the satisfaction after completing the six-week intervention. The TSQ uses three separate scales, whether they felt that the intervention was worth their time (*yes/no*), feelings of self-efficacy (1 (*greatly reduced*) to 5 (*greatly increased*)), and satisfaction with the treatment (1 (*very dissatisfied*) to 5 (*very satisfied*)).

#### Adverse Effects Questionnaire (AEQ [31])

The measure uses a yes/no scale and contains four items and two follow up questions if adverse effects are endorsed. The AEQ is employed to determine whether participants suffered from any adverse effects during the intervention.

### Analysis

As the current study is a pilot of the *Infertility ACTion* program, we were primarily interested in the feasibility of the implemented intervention rather than to determine its overall effectiveness. This aimed to investigate the practicality, viability, and potential challenges associated with the intervention. Therefore, we were most interested in participants’ ratings of utility and suggestions for improving the content and modules. That being said, we also examined their descriptive statistics of the pre- and post-intervention measures looking at means and standard deviations. Pre- and post-intervention comparisons were completed using paired samples t-tests. All data was analyzed using SPSS version 28.0.

## Results

### Participant characteristics

A total of 38 individuals completed the eligibility survey and 28 were found to be eligible as 10 individuals scored too high on the FertiQoL to participate. Of the 28 eligible individuals, 20 agreed to participate. Baseline characteristics of the 20 individuals who agreed to participate are summarized in Table 2. All participants included in our study were from Canada, half from the Province of Ontario. All participants reported identifying as women. Most of the sample identified as being of European origins and being in a committed relationship. Participants' ages ranged from 26 to 41, with an average in the mid-thirties. Participants were of fairly high socioeconomic status, with 70% having a total annual household income > $113,000. The sample of this study was also highly educated: 45% reported their highest level of completed education as a bachelor’s degree, 20% achieved a master's degree, and 10% completed a doctorate. On average, participants experienced a moderate severity of depressive symptoms, with fifty percent of participants scored above the cut-off score of 10, indicating they experienced clinically significant symptoms of depression (Table 2).

**Table 2:** Participant characteristics

Mean age (SD)	34.6 (4.4)
Age range	26–41
Ethnicity	
European origins	75%
Asian origins	10%
African origins	5%
Latin, Central, or South American Origins	5%
Indigenous	5%
Sexual orientation	
Asexual	10%
Bisexual	10%
Heterosexual	75%
Lesbian	5%
Identify as a woman	100%
Province	
Ontario	50%
Alberta	25%
Manitoba	10%
Newfoundland and Labrador	10%
Saskatchewan	5%
Mean time trying (SD) in months	30.9
Time trying range in months	9–97
Education level	
College/ trade school diploma	20%
Some university	5%
Bachelors degree	45%
Master’s degree	20%
Doctorate degree	10%
Mean annual household income	113 000 +
Marital status	
Married	75%
Common law	15%
Single, never married	5%
Engaged	5%
Reproductive health	
Previous pregnancy	40%
Previous early pregnancy loss	40%
Late pregnancy loss or stillbirth	5%
Live birth	15%
Women with polycystic ovarian syndrome (PCOS)	15%
Male factor	15%
Diminished ovarian reserve	35%
Repeated pregnancy loss	5%
Endometriosis	10%
Pelvic inflammatory disease	5%
Uterine abnormalities	10%
Tubal blockage	15%
Unexplained	40%
Fertility treatment	
Current	90%
Ovulation induction	55%
Intrauterine insemination	45%
Egg retrieval	45%
Fresh or frozen embryo transfer	35%
Donor gametes	10%
Mean PHQ-9 score (SD)	11.1 (3.9)
% PHQ-9 > 10	50%
Mean GAD7 score (SD)	10.6 (4.4)
Mean FertiQoL Core score (SD)	44.6 (8.4)
Mean FertiQoL Emotion subscale (SD)	29.2 (12.3)

### Intervention adherence and acceptability

Twenty participants began the Infertility ACTion Program, and 16 completed the entire program and follow-up questionnaire; one participant dropped out after the first week of the intervention, two participants dropped out after the second week, and one participant dropped out after the third week.

Participant responses to the pre-intervention Credibility and Expectancy Questionnaire are reported in Table 3. Most participants endorsed the program as logical and predicted a 50% improvement in symptoms as a result of participating in the program.

**Table 3:** Pre-intervention responses to the credibility and expectancy questionnaire

	M (SD)
How logical does the Infertility ACTion program seem? (1–10)	7.4 (1.1)
How successful do you think the program will be in reducing your symptoms? (1–10)	5.8 (2.0)
How confident would you be in recommending it to a friend? (1–10)	5.9 (2.0)
How much improvement in your symptoms do you think will occur? (1–100)	51.3 (18.5)
How much do you really feel that the program will help you to reduce your symptoms? (1–10)	5.5 (1.8)
How much improvements in your symptom do you really feel will occur? (1–100)	50.0 (22.4)

As shown in Table 4, participants assigned an average helpfulness score of 7.0/10 for the weekly modules and average of 6.6/10 for the weekly homework assignments. They completed an average of 39.6 min of homework each week.

**Table 4:** Weekly Helpfulness Ratings and Self-Reported Homework Completion, M(SD)

Intervention Module	Intervention helpfulness rating (0–10)	Homework helpfulness rating (0–10)	Self-reported minutes of homework
Module 1: values	7.0 (1.4)	6.7 (1.3)	38.6 (24.0)
Module 2: cognitive Defusion	7.2 (1.6)	7.1 (1.6)	45.0 (39.0)
Module 3: present moment focus	7.1 (1.1)	7.1 (0.9)	42.4 (24.4)
Module 4: acceptance	6.7 (1.4)	6.2 (1.4)	38.1 (20.5)
Module 5: self as context	7.1 (1.6)	6.0 (2.3)	42.6 (30.3)
Module 6: committed action	6.8 (1.6)	6.4 (1.6)	30.8 (14.7)

Post-intervention responses to the Treatment Satisfaction Questionnaire are reported in Table 5. Eleven out of 16 participants were “satisfied” or “very satisfied” with the intervention, 14/16 felt it was worth their time and 13/16 would recommend it to a friend and felt that the intervention increased their confidence in managing their symptoms. On the Adverse Effect Questionnaire, no participants endorsed any adverse effects associated with participating in the program, but 4/16 reported experiencing an unwanted event during the intervention. One participant noted that they felt that this unwanted event impacted their ability to engage with the intervention (Table 5).

**Table 5:** Post intervention responses to the Treatment Satisfaction Questionnaire and Adverse Effects Questionnaire

	*N (%)*
*Treatment satisfaction*	
Would you recommend the intervention to a friend?	
Yes	13 (81.3)
No	3 (18.8)
Was the intervention worth your time?	
Yes	14 (87.5)
No	2 (12.5)
How satisfied were you with the intervention?	
Very dissatisfied	1 (6.3)
Dissatisfied	0
Neutral	4 (25)
Satisfied	9 (56.3)
Very satisfied	2 (12.5)
How satisfied were you with the quality of the materials?	
Very dissatisfied	0
Dissatisfied	0
Neutral	2 (12.5)
Satisfied	10 (62.5)
Very satisfied	4 (25)
How has the intervention affected your confidence in managing your symptoms?	
Greatly reduced	0
No change	3 (18.8)
Increased	12 (75)
Greatly increased	1 (6.3)
How has the intervention affected your motivation to seek treatment if needed in the future?	
Reduced	1 (6.3)
No change	4 (25)
Increased	9 (56.3)
Greatly increased	2 (12.5)
*Adverse effects*	
Did you experience any negative effects of treatment?	
Yes	0
No	16 (100)
Did you experience any new psychological symptoms while working through the intervention?	
Yes	1 (6.3)
No	15 (93.8)
Did you experience any unwanted events during the intervention? (e.g., death of a family member)	
Yes	4 (25)
No	12 (75)
Averse events	
Death in family	2 (12.6)
New infertility diagnosis	1 (6.3)
Failed cycle	1 (5.3)
If so, do you feel it negatively impacted your participation, engagement, or benefit from the program?	
Yes	1 (25)
No	3 (75)

### ACT-based psychological processes

Pre-to-post changes on the Multidimensional Psychological Flexibility Inventory are shown in Table 6. Effect sizes associated with changes in psychological flexibility and inflexibility – the two main processes by which ACT is hypothesized to benefit emotional wellbeing – both fell within the medium to large range. Among the dimensions of “flexibility”, changes in “acceptance” were found to be the largest; among the dimensions of “inflexibility”, changes in the “self as context” subscale was largest.

**Table 6:** Pre and Post ACT-based psychological processes

	Pre M(SD)	Post M(SD)	Cohen’s d	Interpretation
Flexibility	3.38 (0.68)	3.92 (0.71)	0.66	Medium to large
Acceptance	2.84 (0.92)	3.69 (0.65)	0.85	Large
Present moment focus	3.77 (0.87)	4.16 (0.67)	0.39	Small to medium
Self as context	3.33 (0.72)	3.7 (0.95)	0.44	Small to medium
Cognitive defusion	2.67 (0.82)	3.28 (0.80)	0.64	Medium
Values	3.86 (0.72)	4.31 (0.95)	0.43	Small to Medium
Committed Action	3.71 (0.20)	4.09 (0.27)	0.39	Small to medium
Inflexibility	3.40 (0.73)	2.86 (0.77)	0.76	Medium to large
Experiential avoidance	4.10 (1.00)	3.61 (0.77)	0.44	Small to medium
Lack of contact with present	3.11 (1.00)	2.37 (0.97)	0.60	Medium
Self as context	3.59 (1.09)	2.84 (0.99)	0.70	Medium to large
Fusion	3.87 (0.99)	3.19 (1.14)	0.46	Small to medium
Lack of contact with values	2.73 (0.78)	2.47 (1.09)	0.25	Small
Inaction	3.09 (0.95)	2.73 (1.16)	0.39	Small to medium

### Mental health outcomes

Table 7 depicts the pre-to-post intervention changes in mental health outcomes. Though changes in depressive and anxious symptoms were in the small to medium range, changes in fertility quality of life fell in the medium range. Of the subscales of the FertiQoL, the “emotional” subscale was associated with the largest effect.

**Table 7:** Pre-to-Post Changes in Mental Health Outcomes

	Pre M(SD)	Post M(SD)	Cohen’s d	Interpretation
PHQ-9	10.3 (4.1)	7.9 (4.8)	0.43	Small to medium
GAD-7	9.9 (4.0)	8.4 (6.0)	0.29	Small
FertiQoL Core	44.6 (8.4)	50.5 (13.2)	0.49	Medium
Emotional	29.2 (12.3)	39.3 (18.9)	0.56	Medium
Mind Body	38.7 (17.7)	41.7 (20.0)	0.20	Small
Relational	71.2 (12.6)	76.3 (13.8)	0.40	Small to medium
Social	44.9 (17.3)	48.5 (16.6)	0.25	Small
RDAS	20.5 (4.1)	20.9 (7.1)	0.09	Small

### Intervention feedback

Qualitative feedback suggests that participants valued the analogies and examples used throughout the program and that the program was specifically tailored to infertility. They also appreciated the convenient format of the program and that the weekly videos were short and that each module ended with a wrap-up summary. Several participants appreciated the mindfulness exercises included in the program.

At the same time, participants recommended several potential changes to the program. Some feedback was contradictory, such as when some participants reported disliking an exercise and the same number of participants report specifically benefiting from that exercise. Similarly, participants liked that the videos were so short but also wanted more examples included and further discussion of numerous topics. Participants feedback can be found in the Additional file 1.

## Discussion

This study pilot tested a self-directed ACT-based intervention adapted for infertility, created by our team, in collaboration with women experiencing infertility. Of the 20 women who enrolled in the pilot, 16 finished all six weeks of the intervention and the follow-up survey, translating to a 20% dropout rate, which is favourable when compared to a recent meta-analysis of online-based trials for depression finding a mean dropout rate across studies of 50% [32]. Research testing self-administered versions of both ACT and CBT for mental health problems unrelated to infertility have observed similarly high dropout rates [33, 34].

ACT which focuses on acceptance, mindfulness, and living a values life, without fighting against what is lost [20], has been shown to improve anxiety, depression, and distress in individuals with advanced cancer, families facing challenging health circumstances, and individuals experiencing chronic disease [35–37]. It is unsurprising that this approach aligns with some of the challenges faced by women dealing with infertility, as it encourages individuals to accept difficult emotions and thoughts while pursuing a meaningful life.

Prior to starting the intervention, participants rated it as highly credible and expected to benefit from the program. Though one might expect a self-directed intervention to have lower credibility or expectancy scores than other modalities [38], scores observed were similar to those seen for in-person ACT-based and CBT-based interventions [39, 40]. Post-intervention satisfaction was also quite high with nearly 90% reporting that they would recommend the program to a friend.

ACT-based interventions have been explored in a self-directed format in various contexts, demonstrating its potential acceptance across therapeutic formats [41]. This suggests that ACT can be delivered effectively though online platforms, making it accessible for self-directed use. This has also been tested in exploring self-directed formats with specific populations like individuals with type 2 diabetes, advanced cancer, and individuals with acquired brain injury [35, 42, 43]. The Infertility ACTion program may be the next targeting intervention for a health population to create accessible and affordable access to evidence based psychological interventions.

Some of the recommended changes from participants would be easy to implement, such as providing more mindfulness exercises and allowing participants to use their own mindfulness recordings. The program could also be made longer (e.g., to eight weeks) to include some of the recommended content. An alternative approach would be to create short supplementary modules on some of the topics identified, such as jealousy and anger.

Future iterations of this program may include changes aimed at improving homework compliance as the average number of minutes spent on homework each week ranged from 38- 45, equating to an average of under seven minutes each day. Other studies have reported homework compliance of closer to 30–45 min a day [44]. Since homework compliance has been found to predict positive psychological outcomes in self-administered and online interventions [45–47], it seems worthwhile attempting to increase the time spent completing homework. One simple tactic, which was recommended by several participants, is to increase the number of reminders to complete the homework. Certain homework assignments may also benefit from some editing; for example, a few participants recommended replacing the “80th birthday” exercise to the “65th birthday” exercise to avoid triggering an existential crisis. Such adjustments may increase homework helpfulness ratings, which was found to be 6.6/10 in the current study. In turn, greater homework completion and satisfaction may increase the effects of the program on mental health.

Though small changes to the Infertility ACTion Program may be warranted, program completion was associated with medium to large changes in both flexibility and inflexibility, the processes by which ACT is believed to benefit well-being. Of the flexibility subscales, *acceptance* and *cognitive defusion* exhibited the largest changes; of the inflexibility subscales, *lack of contact with the present moment* and *self as context* exhibited the largest changes. In terms of mental health outcomes, a medium effect was observed for overall fertility quality of life, with the emotional subscale showing the largest increase. In contrast, declines in depressive and anxious symptoms were small. This finding is consistent with the fact that while ACT is an intervention recognized by APA Division 12 for use with chronic pain, it is not currently recognized as having strong evidence for use with depression or anxiety [48]. Indeed, the focus of ACT is not on symptom reduction but rather on symptom acceptance and the commitment to live one’s life to the fullest despite one’s symptoms. Thus, it is perhaps not surprising that improvements in flexibility are accompanied by an improvement in quality of life despite only small changes in symptoms of psychopathology.

The findings of this study should be considered in light of a few limitations. First, the sample size was small and better educated than the population average, perhaps limiting the generalizability of our findings. Second, module and homework completion were assessed via self-report and are therefore subject to inaccuracies. Third, only short-term mental health outcomes were assessed; it is possible that mental health may have continued to improve as participants continued to implement the skills they learned throughout the program. Finally, the current study consisted of a one-arm within-person study, which makes it difficult to differentiate the effects of the intervention from the effects of the passage of time. A randomized controlled trial will be better suited to addressing this issue in the future.

## Conclusion

Infertility currently affects one in six women and is becoming increasingly prevalent as more women choose to delay childbearing. For many, it will be the most upsetting experience of their lives. Currently, adapted interventions for infertility-related distress have had limited success in clinically significant reductions in symptomology for women with infertility. ACT is an evidence-based intervention that has received limited exploration for women with infertility. This pilot has demonstrated that an ACT-based self-directed intervention is feasible with this population; more rigorous research and the above-mentioned changes are needed to help clarify the efficacy of this intervention.

### Supplementary Information

**Additional file 1:** Intervention Feedback.

## Data Availability

The datasets generated and/or analyzed during the current study are not publicly available to protect the identities of the participants involved. Anonymized data supporting the findings of this study are available from the corresponding author, upon reasonable request.

## Footnotes

1 Throughout this manuscript women will be used to refer to individuals attempting to conceive and possessing a uterus. It is important to consider that not all individuals matching this description will identify as women and may have unique experiences of distress.
