# Supplementary material for: Acceptance and commitment therapy adapted for women with infertility: a pilot study of the Infertility ACTion program
Source: Reprod Health. 2024 Apr 4;21:43. doi: 10.1186/s12978-024-01766-5 (PMC10996141; doi:10.1186/s12978-024-01766-5)
Supplement: Supplementary file 1 — Additional file 1: Intervention Feedback. [file 12978_2024_1766_MOESM1_ESM.docx]

| Supplementary Material  Intervention Feedback | |
| --- | --- |
| Module-Specific Feedback | |
| Module 1: Values | Recommendations  Acknowledge culture may play a role in values (1)  Replace the 80^th^ birthday exercise with something else (2)  Include more values exploration (1)  Remove discussion of choice-points (1) |
|  | Positive Feedback  Liked the content (1)  Liked the 80^th^ birthday party exercise (2)  Liked the discussion of choice-points (2) |
| Module 2: Cognitive defusion | Recommendations  Make this the first module (1)  Replace the discussion on cognitive defusion with an exploration the meaning of the thoughts (1)  Replace the image of the pink elephant (too baby-related) (1)  Experienced trouble consistently practicing this skill (2) |
|  | Positive Feedback  Liked the “leaves on a stream” analogy (3)  Appreciated discussion about thoughts not being reality (1) |
| Module 3: Present moment focus | Recommendations  Don’t make assumptions about the relational nature of fertility (1)  Allow participants to complete their own mindfulness exercise recordings/ add more exercises (2)  Include more reminders to engage in mindfulness practice (2) |
|  | Positive Feedback  Enjoyed the mindfulness exercises (7) |
| Module 4: Acceptance | Recommendations  Don’t make assumptions about women with infertility experiencing painful emotions (1) |
| Module 5: Self as Context | Recommendations  Apply a narrative technique to exploring identity (1)  Include more reminders to complete the homework (1)  Found this module difficult in separating what has happened to them from their idea of themselves (1) |
|  | Positive Feedback  Appreciated the “continuous you” exercise (1) |
| Module 6: Committed Action | Found it difficult to engage with content of the module while getting difficult news (e.g., negative pregnancy test) (1) |
| Feedback on the Program as a Whole | |
| Program Content | Recommendations  Add more examples throughout (1)  Add journal/diary prompts for reflection (2)  Provide more variety in the mindfulness exercises (1)  Add more content on the impact of infertility on mental health (1)  Include a module on the nervous system (1)  Include examples for trying to conceive without a partner (1)  Include examples of fertility treatments for genetic reasons (1)  Add a discussion of financial stress due to infertility (1)  Include more content about jealousy and anger (2)  Include more discussion on how to manage disappointment (1)  Include more content related to self-compassion (1)  Positive Feedback  Includes infertility-specific content (3)  Enjoyed the use of examples (1) |
| Program Delivery | Recommendations  Enhance the quality of image and sound (1)  Remove blank screens during meditation or reflection exercises (1)  Provide the modules in podcast format (1)  Include a longer break at the end of meditations (1)  Include additional reminders for homework completion (5)  Include subtitles to the videos (1)  Show the person who is speaking (1)  Make the voiceover faster (1)  Include the support of a psychologist (1)  Increase the program length (2)  Positive Feedback  Videos were short (3)  Summaries at the end of each module were helpful (4) |
